# CT delta-radiomics predicts the risks of blood transfusion and massive bleeding during spinal tumor surgery

**DOI:** 10.1186/s40644-025-00900-1

**Published:** 2025-06-22

**Authors:** Suwei Liu, Yali Li, Shuai Tian, Chenyu Jiang, Ming Ni, Ke Xu, Feng Wei, Huishu Yuan

**Affiliations:** 1 Department of Radiology, Peking University Third Hospital, 49 Huayuan N Rd, Haidian District, Beijing, China; 2 Department of Orthopaedic Surgery, Peking University Third Hospital, 49 Huayuan N Rd, Haidian District, Beijing, China

**Keywords:** Delta-radiomics, Spinal tumor, Logistic regression, Blood transfusion, Intraoperative bleeding

## Abstract

**Background:**

Intraoperative bleeding is a serious complication of spinal tumor surgery. Preoperative identification of patients at high risk of intraoperative blood transfusion (IBT) and intraoperative massive bleeding (IMB) before spinal tumor resection surgery is difficult but critical for surgical planning and blood management. This study aims to develop and validate delta radiomics prediction models for IBT and IMB in spinal tumor surgery.

**Methods:**

Patients diagnosed with spinal tumors who underwent spinal tumor resection surgery were retrospectively recruited. CT, CTE, delta, and clinical models based on CT native phase, CT arterial phase images, and clinical factors were constructed using 10-fold cross-validation and logistic regression (LR), random forest (RF), and support vector machine (SVM) in the training cohort. Receiver operating characteristic (ROC) curves, integrated discrimination improvement (IDI), accuracy, sensitivity, specificity, positive predictive value, and negative predictive value were used to evaluate and compare the diagnostic performance of these models.

**Results:**

231 patients were randomly divided into training (*n* = 161) and test (*n* = 70) cohorts, comprising 146 IBT and 85 no-IBT patients, 35 IMB and 196 no-IMB patients, respectively. The delta model performed best in predicting IBT and IMB risk, with better predictive ability than the clinical model (IDI = 0.11–0.13 for IBT, and IDI = 0.02–0.08 for IMB, *p* < 0.05, respectively). Calibration curves indicated that the predicted probabilities of IBT and IMB in the model did not differ significantly from the actual probabilities (*p* > 0.05).

**Conclusion:**

The CT delta model we constructed may be a valuable tool to improve risk stratification before spinal tumor surgery, thus contributing to preoperative planning and improving patient prognosis.

**Trial registration:**

Retrospectively registered (M2020435).

**Supplementary Information:**

The online version contains supplementary material available at 10.1186/s40644-025-00900-1.

## Background

Spinal tumors often require surgical resection, especially when there is nerve compression, spinal instability, and intractable pain [1, 2]. However, spinal tumor surgeries are frequently complicated by intraoperative bleeding, due to the spine’s complex anatomy and rich vascular supply [3, 4].

To manage potential blood loss, clinicians typically prepare blood in advance. However, this practice can lead to either over-preparation or under-preparation, resulting in surgical delays, inefficient use of blood resources, and challenges in blood bank management [5, 6]. Moreover, excessive transfusion of allogeneic blood during surgery is associated with increased postoperative risks, such as infections, delirium, venous thromboembolism, myocardial infarction, and even mortality [7]. Therefore, preoperative identification of the risk of intraoperative blood transfusion (IBT) and intraoperative massive bleeding (IMB) is critical for patient prognosis, surgical planning, and blood management.

There are several preoperative methods for assessing the blood supply to the tumor and the peritumoral vascular distribution, such as contrast-enhanced CT and dynamic contrast-enhanced magnetic resonance imaging (DCE-MRI). These methods often involve observing numerical differences or signal changes in different scanning phases. However, in addition to being more subjective, they are often limited by bone disturbances or spatial heterogeneity [8–10]. Digital subtraction angiography, an invasive test, is often used in conjunction with treatments such as vascular embolization [11, 12]. Therefore, the precision assessment of the blood supply of tumors has become a challenge.

Radiomics, which uses advanced imaging analysis powered by artificial intelligence, can quantitatively analyze biomedical images to extract valuable pathophysiological information [13, 14]. Specifically, delta radiomics, which tracks relative changes in imaging features over time, has shown potential benefits for several clinical endpoints in oncology, such as differential diagnosis, prognosis and prediction of treatment response, and evaluation of side effects [15, 16]. In previous studies, Sushentsev et al. [17] found that delta radiomics could monitor the pathological progression of prostate cancer. Chen et al. [18] quantified image features using delta radiomics to measure differences before and after immunotherapy for metastatic melanoma. However, few studies have used delta radiomics to quantify tumor blood supply to predict IBT and IMB.

Thus, we aim to develop and validate predictive models that combine delta radiomics with clinical variables to preoperatively identify the risk of IBT and IMB in spinal tumor surgery.

## Materials and methods

This retrospective study was approved by our Hospital Institutional Review Board and written informed consent was waived by the Declaration of Helsinki (M2020435).

### Study patients

Patients with spinal tumors who underwent surgery at our hospital between January 2013 and December 2023 were included in this study. The inclusion criteria were as follows: pathologically confirmed spinal tumor; complete preoperative CT native phase and CT arterial phase images; and complete clinical and surgical information. The exclusion criteria were as follows: poor image registration; history of multiple spinal surgeries; not performed open surgery; and autologous blood transfusion. The clinical and imaging data of the patients were collected from medical records and imaging archives.

### Clinical characteristics and radiographic assessment

Clinical variables included age, sex, preoperative albumin and hemoglobin levels, platelet count, preoperative embolization status (yes or no), tumor type (primary or secondary), and pathological classification of biopsy specimens (benign or malignant). All clinical indicators were measured before surgery, with the values being recorded as close as possible to the day of the operation to minimize any temporal discrepancies. Radiographic characteristics included tumor location (lumbosacral or other), number of affected segments (single or multiple), and tumor size (axial maximum long and vertical short diameter). Radiographic assessments were carried out using preoperative imaging performed within two weeks before surgery. Three readers (with 12, 7, and 5 years of musculoskeletal imaging experience) independently reviewed all images in a blinded manner to assess radiographic characteristics. Any disputes were resolved by a majority vote.

The first primary outcome was IBT, which was defined as the intraoperative administration of one or more units of allogeneic blood based on a joint decision by the anesthesiologist and surgeon. The second primary outcome was IMB, which was defined as an estimated blood loss of ≥ 2500 mL [5]. Intraoperative blood loss can be estimated by an anesthesiologist using the outputs from the suction canister and irrigation volumes recorded at the end of surgery. Alternatively, the theoretical total blood loss can be calculated using the following formula: estimated blood loss (mL) = (preoperative or estimated hematocrit - measured hematocrit) / preoperative or estimated hematocrit × body weight (kg) × 7% × 1000.

### CT study protocols

During this study, we utilized spine (cervical, thoracic, lumbar) scan protocols and selected CT native phase and arterial phase images. The imaging parameters are listed in Supplementary Material 1.

### Image registration and segmentation

**Fig. 1:**
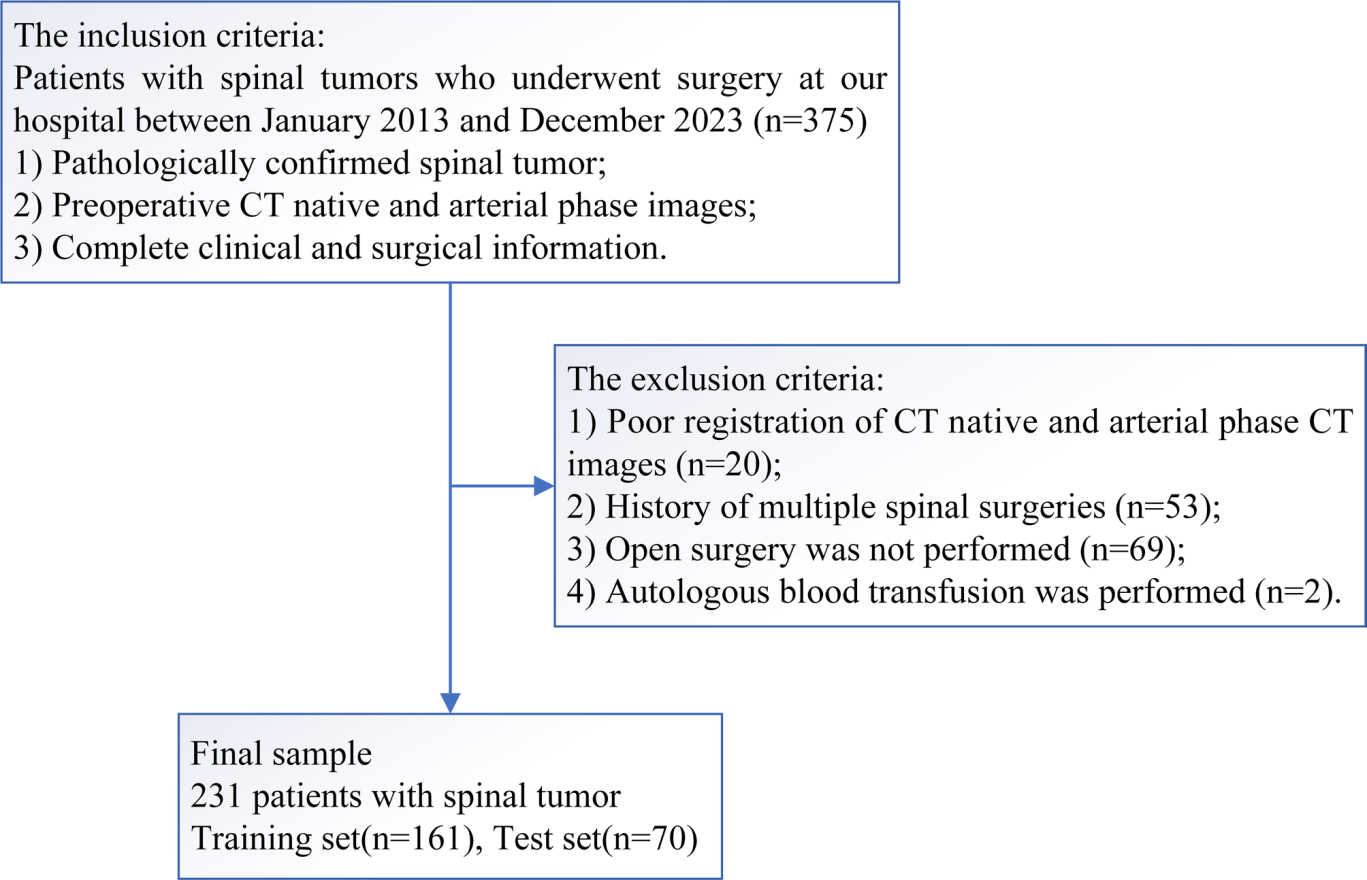
Flowchart of patient inclusion

The radiomics feature extraction workflow is shown in Fig. 1. The CT native phase and arterial phase images had identical positioning, and the start and end points were consistent, allowing for automatic image registration. Using ITK-SNAP software (version 3.8.0; the University of Pennsylvania and University of Utah). We defined the Region of Interest (ROI) to encompass the entire tumor mass, including both the osseous and extraosseous components, based on preoperative CT imaging. Reader 1 simultaneously manually delineated the tumors on axial CT native phase and arterial phase images and repeated segmentation twice within 1 week to assess feature stability. Reader 2 randomly selected 60 patients, initially performed independent segmentation, and then performed simultaneous segmentation to verify the consistency of the image features observed during independent and simultaneous segmentation. If the difference was ≥ 5%, reader 3 redefined the tumor boundaries. Radiomics features were evaluated using the intra-class correlation coefficient (ICC), and features with an ICC > 0.80 were retained.

### Radiomics features extraction

Before radiomics features were extracted, the images were standardized by adjusting the standard deviation to 100, resampling the volumes of interest on CT to uniform 1 × 1 × 1 mm voxels using linear interpolation, and preprocessing the CT images with all features set to a bin width of 10 HU. Radiomics features were extracted using the PyRadiomics Python package (version 2.2.0) [19]. Radiomics features encompass the grey level co-occurrence matrix (GLCM), first-order grey level run length matrix (GLRLM), grey level size zone matrix (GLSZM), grey level dependence matrix (GLDM), neighboring grey tone difference matrix (NGTDM), and shape from the segmentation regions for each MRI. Specifically, GLCM measures spatial interdependencies between voxels that display differing grey levels; GLRLM identifies the distribution of consecutive voxels that share the same grey level values throughout the image; GLSZM represents the distribution of size zones within the image; GLDM counts neighboring voxels with specific grey level differences; NGTDM calculates the grey level value discrepancy between a voxel and its surrounding counterparts’ mean values. The first-order technique outlines the voxel intensity distribution in the image region designated by the mask using standard statistical measures, whereas shape features relate to the geometric characteristics of the specific area, which entail factors such as volume, surface area, compactness, sphericity, and other relevant aspects [20].

The delta radiomics features were defined as the relative net change of radiomics features between arterial phase images (a) and native phase images (b): i.e., delta radiomics features = (a - b) / b [21].

### Feature selection and classification methods

To avoid multicollinearity and minimize the risk of overfitting when dealing with high-dimensional radiomics features, each radiomics feature was normalized [Z=(x-mean)/standard deviation] and standardized, and the batch effects were then removed. Next, Student’s t-tests of the features were performed, and all features with *p* < 0.05 were retained. To address multicollinearity among the features, we conducted a redundancy analysis (using Pearson or Spearman methods) and eliminated features with a correlation coefficient > 0.65.

For logistic regression (LR), the least absolute shrinkage and selection operator (LASSO) was employed for feature selection, as it imposes a penalty on the number of features and helps mitigate multicollinearity. The final selected features were then used to refit a non-penalized LR model to better interpret the linear relationships between features and outcomes. For random forest (RF), recursive feature elimination was applied to select important features based on the ability of the model to handle non-linear interactions and feature importance. Finally, LR, RF, and support vector machine (SVM) classification algorithms, along with 10-fold cross-validation, were used to identify the most predictive features from the training cohort.

### Prediction models development

Univariate and multivariate analyses were conducted to identify the clinical and radiographic factors associated with IBT and IMB during spinal tumor surgery. To reduce the uncertainty of random grouping and balance the accuracy and complexity of the models, models were constructed using LR, RF, and SVM for the training cohort. The radiomics score of each sample was then calculated based on the regression model coefficients and the importance-ranked radiomics features. Ultimately, CT, CTE, and delta models were developed and combined with clinical models to predict the risks of IBT and IMB for spinal tumor patients, and the corresponding nomograms were constructed.

### Statistical analysis

Statistical analyses were performed using SPSS software (Version 26.0; IBM et al., N.Y., USA) and R software (version 4.4.0; https://www.r-project.org). Continuous variables were summarized using mean ± standard deviation, while categorical variables were expressed as frequencies and percentages. Continuous variables (Age, HB, and ALB) were compared using the independent samples t-test or the Mann–Whitney U test. Categorical variables were compared using the chi-square test or the Fisher test. To identify independent predictors of intraoperative transfusion and massive intraoperative bleeding, we conducted univariate and multivariate logistic regression analyses. Variables with a *p*-value < 0.05 in univariate analysis were included in the multivariate model to determine independent risk factors. Diagnostic efficacy was evaluated using the area under the receiver-operating characteristic curve (AUC), integrated discrimination improvement (IDI), accuracy, sensitivity, specificity, positive predictive value, and negative predictive value. The Youden index (i.e., maximizing “sensitivity + specificity − 1”) was used to optimize the cutoff point for dichotomizing model predictions into binary categories. Calibration curves and a decision curve analysis were used to assess the clinical utility of the nomograms of the validation cohort. The packages in R software involved in this study are shown in Supplementary Material 2.

## Results

### Patients characteristics

**Fig. 2:**
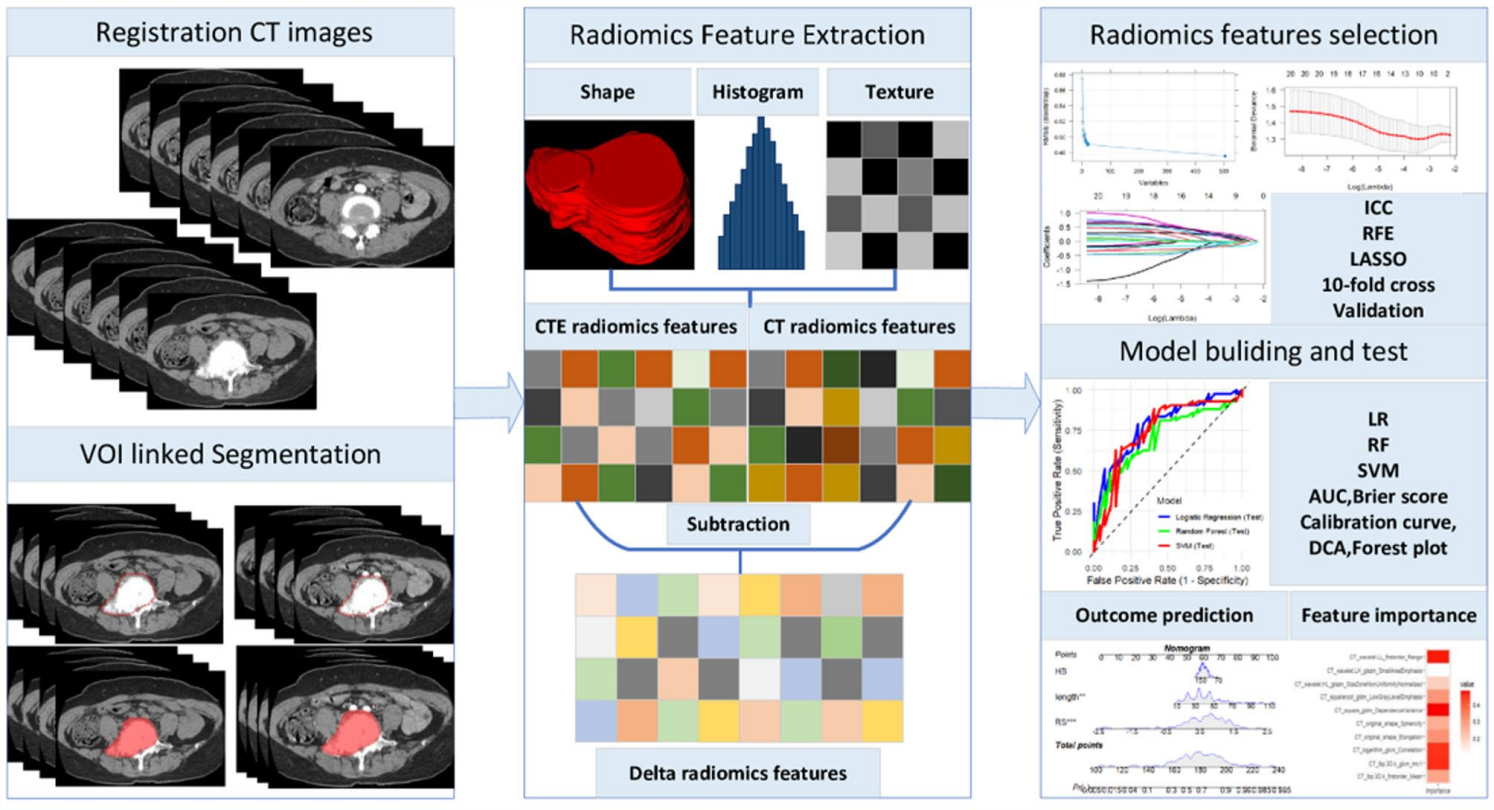
Workflow for VOI segmentation, radiomics feature extraction, model building, and downstream analysis. AUC, area under the receiver operating characteristic curve; DCA, decision curve analysis; ICC, intraclass correlation coefficient; RFE, recursive feature elimination; LASSO, least absolute shrinkage and selection operator; LR, logistic regression; RF, random forest; SVM, support vector machine; VOI, volume of interest

Of the 375 patients, 144 were excluded (Fig. 2). The final study cohort comprised 231 patients, including 123 males and 108 females with an age range of 10–79 years. Among the patients, there were 85 no-IBT patients (median age 46 years; range 12–79 years) and 146 IBT patients (median age 42 years; range 10–79 years). Additionally, there were 35 IMB patients (median age 40 years; range 15–63 years) and 196 no-IMB patients (median age 45 years; range 10–79 years). The clinical characteristics of patients in the training and testing sets are summarized in Supplementary Material 3.

### IBT prediction models

In total, 1,688 × 3 radiomics features were obtained. Ultimately, 10 features were selected to develop the CT radiomics model, comprising 10 texture features (1 from GLCM, 1 from GLRLM, 4 from GLSZM, and 4 from GLDM), 8 features were selected to develop the CTE radiomics model, comprising 10 texture features (2 from GLCM, 2 from GLSZM, 1 from GLDM), 2 first-order features, and 1 shape feature, and 12 features were selected to develop the delta radiomics model, comprising 10 texture features (3 from GLCM, 3 from GLRLM, 2 from GLSZM, 1 from GLDM, and 1 from NGTDM) and 2 first-order features. The radiomics feature selection process and correlation analysis between radiomics features and IBT are presented in Supplementary Material 4.

**Fig. 3:**
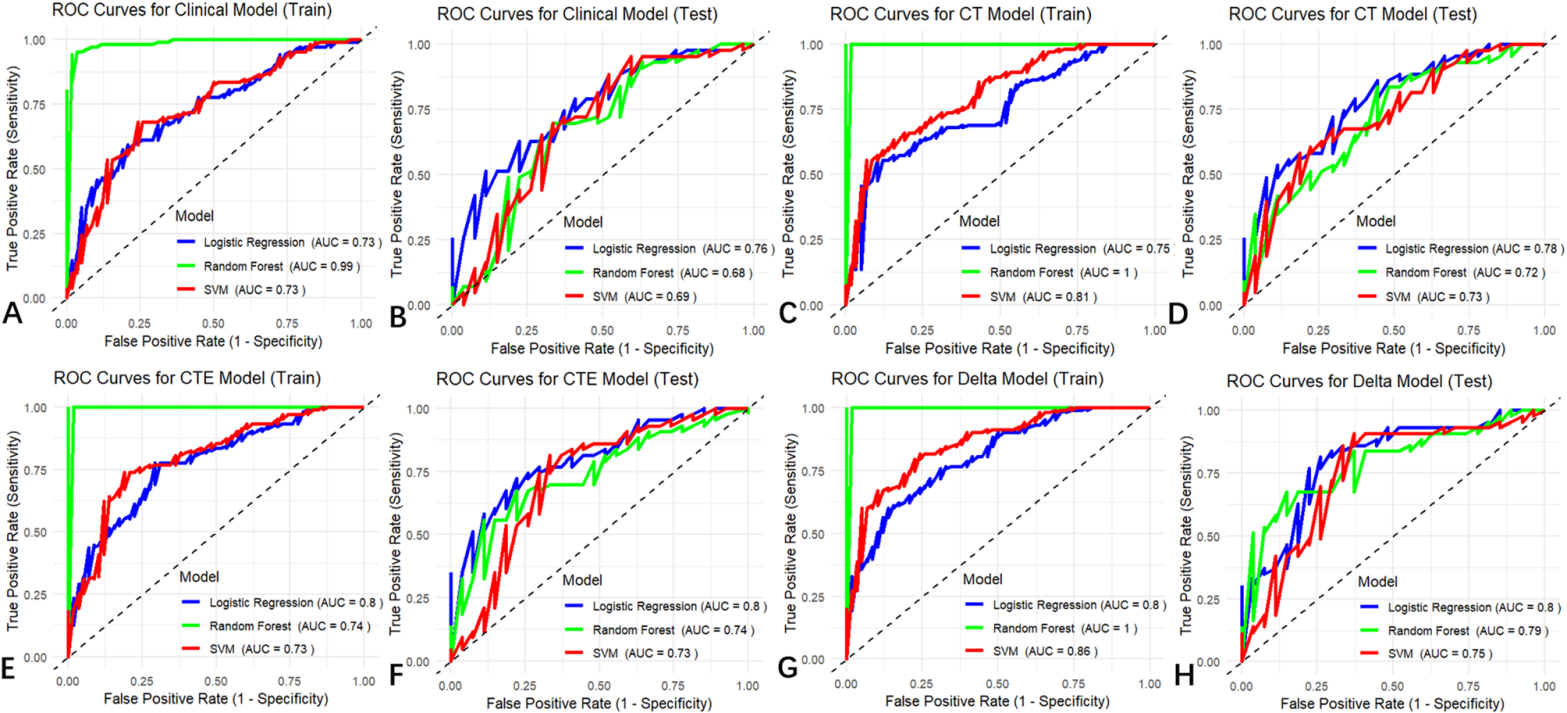
Assessment of models for the ability to predict intraoperative transfusion. ROC curves of clinical, CT, CTE, and delta models are used to predict intraoperative transfusion in the training (**A**, **C**, **E**, **G**) and test sets (**B**, **D**, **F**, **H**). ROC, receiver operating characteristic

The axial maximum long and vertical short diameter of spinal tumors were used as predictors for constructing the clinical model for predicting IBT (Table 1), which was combined with the radiomics models, resulting in the CT, CTE, and delta models, respectively. The results, including the performance of the LR, RF, and SVM classifiers for the training and test sets of clinical, CT, CTE, and delta models are listed in Table 2 and Fig. 3. The results indicated that the CT, CTE, and delta models all had better performance than the clinical model. In particular, the delta model showed the best performance and outperformed the clinical model (IDI = 0.12–0.13, *p* < 0.05) (Table 3). The AUC values of the LR, RF, and SVM classifiers of the training set of the delta model were 0.80, 1.0, and 0.86, respectively, and those for the test set were 0.80, 0.79, and 0.75, respectively.

**Table 1:** Logistic regression analysis of variables for their association with intraoperative transfusion in patients in the training set

Characteristic	Univariable Analysis		Multivariable Analysis	
	OR	*P* Value	OR	*P* Value
Age (y) *	0.99(0.97–1.01)	0.35		
Sex(Male vs. Female)	1.01(0.52–1.92)	0.99		
HB level (U/L) *	0.98(0.97–1.01)	0.02		
ALB level (U/L)	1.00(0.92–1.09)	0.94		
PLT level (U/L)	1.01(0.99–1.04)	0.98		
PE(Yes vs. No)	2.03(0.90–4.90)	0.13		
Tumor type(Primary vs. Metastatic)	1.30(0.59–2.98)	0.66		
Pathological type(Benign vs. malignant)	1.65(0.87–3.17)	0.17		
Tumor location(Lumbosacral vs. others)	1.16(0.51–2.77)	0.90		
Tumor involved segment(Single vs. multiple)	2.26(1.17–4.46)	0.02		
Maximum long diameter (mm) *	1.06(1.03–1.09)	<0.001	1.04(1.01–1.07)	0.001
Vertical short diameter (mm) *	1.05(1.02–1.07)	<0.001	1.03(1.00-1.06)	0.01
RS	4.01(2.06–9.28)	<0.001	4.2(2.10–9.31)	<0.001

Note.—Data in parentheses are 95% CIs. HB = Hemoglobin, ALB = albumin, PLT = platelet, PE = Preoperative embolization, RS = Radiomics score, OR = odds ratio

* Continuous variables (Age, HB and ALB) were compared using the independent samples t-test or the Mann–Whitney U test

**Table 2:** Diagnostic performance of the CT model, cte model and Delta model for predicting intraoperative transfusion

Models	classifiers	Sensitivity	Specificity	Accuracy	PPV	NPV	AUC
Training set (*n* = 161)							
Clinical Model	LR	0.38	0.83	0.66	0.55	0.70	0.73(0.65–0.81)
	RF	0.95	0.95	0.95	0.92	0.97	0.99(0.98-1)
	SVM	0.28	0.90	0.68	0.62	0.69	0.73(0.65–0.81)
CT Model	LR	0.41	0.76	0.70	0.63	0.72	0.75(0.67–0.82)
	RF	1.00	1.00	1.00	1.00	1.00	1.00(1.00–1.00)
	SVM	0.14	1.00	0.69	1.00	0.67	0.81(0.74–0.88)
CTE Model	LR	0.45	0.85	0.71	0.63	0.73	0.77 (0.70–0.84)
	RF	1.00	1.00	1.00	1.00	1.00	1.00(1.00–1.00)
	SVM	0.36	0.97	0.75	0.88	0.73	0.80(0.74–0.88)
Delta Model	LR	0.52	0.86	0.74	0.68	0.76	0.80(0.74–0.87)
	RF	1.00	1.00	1.00	1.00	1.00	1.00(1.00–1.00)
	SVM	0.41	0.94	0.75	0.80	0.74	0.86(0.80–0.91)
Test set (*n* = 70)							
Clinical Model	LR	0.41	0.91	0.71	0.73	0.71	0.76(0.65–0.87)
	RF	0.44	0.74	0.63	0.52	0.68	0.68(0.54–0.82)
	SVM	0.37	0.95	0.73	0.83	0.71	0.69(0.55–0.83)
CT Model	LR	0.41	0.88	0.70	0.69	0.70	0.78 (0.67–0.89)
	RF	0.44	0.88	0.71	0.71	0.72	0.72 (0.60–0.84)
	SVM	0.04	1.00	0.63	1.00	0.62	0.73 (0.60–0.85)
CTE Model	LR	0.44	0.86	0.70	0.67	0.71	0.80(0.70–0.90)
	RF	0.48	0.81	0.68	0.62	0.71	0.74(0.62–0.86)
	SVM	0.18	0.93	0.64	0.63	0.65	0.73(0.60–0.86)
Delta Model	LR	0.41	0.93	0.73	0.79	0.71	0.80(0.70–0.91)
	RF	0.44	0.86	0.70	0.67	0.71	0.79(0.68–0.90)
	SVM	0.30	0.93	0.69	0.73	0.68	0.75(0.63–0.88)

Note.—Data in parentheses are 95% CIs. PPV = positive predictive value, NPV = negative predictive value, AUC = area under the receiver operating characteristic curve, LR = logistic regression, RF = Random Forest, SVM = Support Vector Machine

**Table 3:** Integrated discrimination improvement of the values of the training and testing sets in CT model, CTE model and delta model compare to clinical model

Models	classifiers	Training Set(*n* = 161)	*P* Value*	*P* Value†	Test Set(*n* = 70)	*P* Value*	*P* Value†
**Intraoperative transfusion**							
CT Model	LR	0.03(0.00-0.07)	0.03	0.86	0.02(0.00-0.06)	0.42	0.83
	RF	0.12(0.08–0.17)	0.001	0.05	0.05(0.04–0.15)	0.30	0.58
	SVM	0.02(0.00-0.06)	0.04	0.23	0.03(0.02–0.08)	0.38	0.65
CTE Model	LR	0.08(0.03–0.13)	0.001	0.20	0.05(0.03–0.13)	0.002	0.44
	RF	0.11(0.07–0.16)	0.001	0.05	0.05(-0.07-0.18)	0.44	0.24
	SVM	0.06(0.02–0.11)	0.003	0.36	0.001(-0.06-0.01)	0.09	0.75
Delta Model	LR	0.13(0.07–0.19)	0.001	0.05	0.12(0.06–0.18)	0.001	0.50
	RF	0.13(0.07–0.17)	0.000	0.05	0.13(0.07–0.18)	0.001	0.04
	SVM	0.12(0.06–0.17)	0.001	0.07	0.11(0.05–0.15)	0.001	0.43
**Intraoperative massive bleeding**							
CT Model	LR	-0.03(-0.06-0.01)	0.17	0.31	0.01(-0.04-0.07)	0.68	0.61
	RF	-0.00(-0.02-0.02)	0.96	1	-0.03(-0.11-0.04)	0.40	0.47
	SVM	-0.09(-0.16–0.02)	0.006	0.02	-0.10(-0.21-0.01)	0.07	0.73
CTE Model	LR	0.00(-0.01-0.02)	0.67	0.87	0.01(-0.01-0.02)	0.39	0.36
	RF	0.01(-0.02-0.03)	0.52	1	0.03(-0.06- 0.07)	0.08	0.48
	SVM	0.00(-0.02-0.01)	0.85	0.14	0.07(-0.02-0.17)	0.01	0.86
Delta Model	LR	0.08(0.02–0.13)	0.007	0.20	0.05(-0.01-0.08)	0.02	0.28
	RF	0.02(-0.01-0.04)	0.045	1	0.03(-0.02- 0.06)	0.03	0.51
	SVM	0.07(0.01–0.14)	0.03	0.001	0.03(-0.01-0.04)	0.04	0.37

Note.—Models are named after feature type. Data are the values of integrated discrimination improvement, and data in parentheses are 95% CIs. All image phases were used, unless otherwise indicated. LR, logistic regression; RF, random forest; SVM, support vector machine

* P value for integrated discrimination improvement

**†** P value for the DeLong test

### IMB prediction models

Similarly, 1,688 × 3 radiomics features were obtained. Ultimately, 4 features were selected to develop the CT radiomics model, comprising 1 texture features (1 from GLSZM), 2 first-order features and 1 shape feature, 6 features were selected to develop the CTE radiomics model, comprising 3 texture features (3 from GLSZM) and 3 first-order features, and 10 features were selected to develop the delta radiomics model, comprising 9 texture features (1 from GLCM, 1 from GLSZM, 3 from GLDM, and 4 from NGTDM) and 1 first-order features. The radiomics selection process and correlation analysis between radiomics features and IMB are presented in Supplementary material 5.

**Fig. 4:**
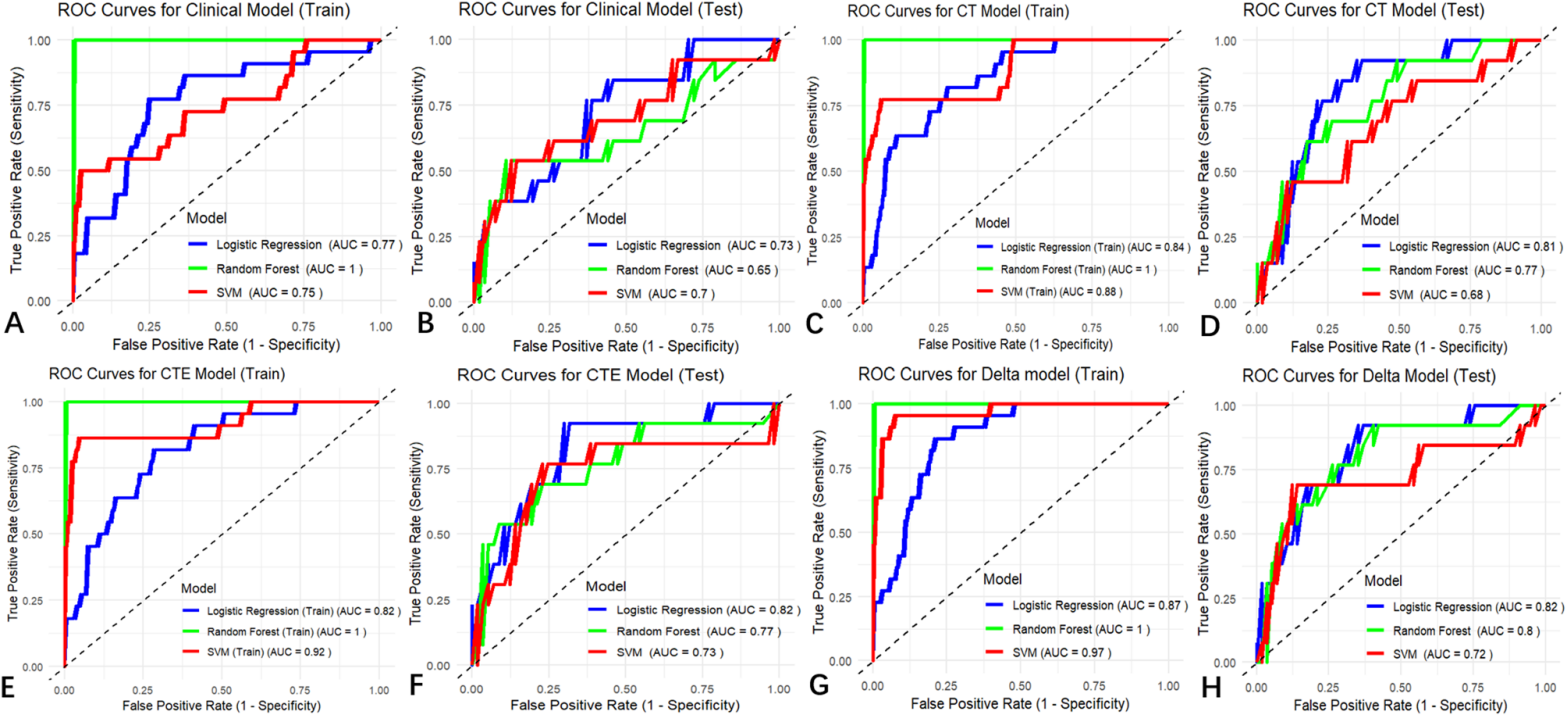
Assessment of models for the ability to predict intraoperative massive bleeding model. ROC curves of clinical, CT, CTE, and delta models are used to predict intraoperative massive bleeding in the training (**A**, **C**, **E**, **G**) and test sets (**B**, **D**, **F**, **H**). ROC, receiver operating characteristic

The patients’ age, platelet count, and tumor axial maximum long diameter were used as predictors for constructing clinical model for predicting IMB (Table 4), which was combined with the radiomics models, resulting in the CT, CTE, and delta models, respectively. The results, including the performance of the LR, RF, and SVM classifiers for the training and test sets of clinical, CT, CTE and delta models are listed in Table 5; Fig. 4. The results indicated that the CT model exhibited slightly lower performance compared to the clinical model. The CTE model demonstrated comparable performance to the clinical model. The delta model showed the best performance, and outperformed the clinical model (IDI = 0.02–0.03, *p* < 0.05) (Table 3). The AUC values of the LR, RF, and SVM classifiers of the training set of the delta model were 0.87, 1.0, and 0.97, respectively, and those of the test set were 0.82, 0.80, and 0.72, respectively.

**Table 4:** Logistic regression analysis of variables for their association with intraoperative massive bleeding in patients in the training set

Characteristic	Univariable Analysis		Multivariable Analysis	
	OR	*P* Value	OR	*P* Value
Age (y) *	0.97(0.95-1.00)	0.045	0.97(0.94-1.00)	0.015
Sex(Male vs. Female)	1.20(0.48–3.07)	0.87		
HB level (U/L) *	0.99(0.97–1.02)	0.86		
ALB level (U/L) *	1.03(0.92–1.14)	0.74		
PLT level (U/L) *	1.00(0.99-1.00)	0.04	0.99(0.98-1.00)	0.045
PE(Yes vs. No)	0.98(0.30–2.71)	1		
Tumor type(Primary vs. Metastatic)	0.32(0.05–1.18)	0.20		
Pathological type(Benign vs. malignant)	1.08(0.44–2.73)	0.99		
Tumor location(Lumbosacral vs. others)	1.80(0.59–4.88)	0.41		
Tumor involved segment(Single vs. multiple)	2.17(0.87–5.57)	0.15		
Maximum long diameter (mm) *	1.05(1.03–1.08)	0.004	1.03(0.99–1.07)	0.006
Vertical short diameter (mm) *	1.03(1.01–1.06)	0.04		
RS	4.01(2.06–9.28)	<0.001	3.24(0.98–12.36)	<0.001

Note.—Data in parentheses are 95% CIs. HB = Hemoglobin, ALB = albumin, PLT = platelet, PE = Preoperative embolization, RS = Radiomics score, OR = odds ratio

* Continuous variables (Age, HB and ALB) were compared using the independent samples t-test or the Mann–Whitney U test

**Table 5:** Diagnostic performance of the CT model, cte model and Delta model for predicting intraoperative massive bleeding

Models	classifiers	Sensitivity	Specificity	Accuracy	PPV	NPV	AUC
Training set (*n* = 161)							
Clinical Model	LR	0.99	0.18	0.88	0.88	0.67	0.77(0.66–0.88)
	RF	1.00	1.00	1.00	1.00	1.00	1.00(1.00–1.00)
	SVM	1.00	0.23	0.89	0.89	1.00	0.75(0.62–0.87)
CT Model	LR	0.96	0.18	0.86	0.88	0.44	0.84 (0.76–0.92)
	RF	1.00	1.00	1.00	1.00	1.00	1.00 (1.00–1.00)
	SVM	1.00	0.09	0.88	0.87	1.00	0.88 (0.80–0.97)
CTE Model	LR	0.98	0.18	0.87	0.88	0.57	0.82 (0.73–0.91)
	RF	1.00	1.00	1.00	1.00	1.00	1.00 (1.00–1.00)
	SVM	1.00	0.18	0.89	0.88	1.00	0.92 (0.84-1.00)
Delta Model	LR	0.97	0.23	0.87	0.89	0.56	0.87 (0.81–0.93)
	RF	1.00	1.00	1.00	1.00	1.00	1.00 (1.00–1.00)
	SVM	1.00	0.32	0.91	0.90	1.00	0.97 (0.93-1.00)
Test set (*n* = 70)							
Clinical Model	LR	0.96	0.23	0.83	0.85	0.60	0.73(0.58–0.88)
	RF	0.96	0.31	0.84	0.86	0.67	0.65(0.45–0.85)
	SVM	0.96	0.23	0.83	0.85	0.60	0.70(0.52–0.89)
CT Model	LR	0.95	0.15	0.80	0.83	0.40	0.81 (0.70–0.93)
	RF	0.95	0.15	0.80	0.83	0.40	0.77 (0.63–0.91)
	SVM	0.98	0.15	0.83	0.84	0.67	0.68 (0.51–0.86)
CTE Model	LR	0.97	0.31	0.84	0.86	0.67	0.82 (0.70–0.95)
	RF	0.97	0.15	0.81	0.83	0.50	0.77 (0.60–0.93)
	SVM	1.00	0.00	0.81	0.81	0.00	0.73 (0.55–0.92)
Delta Model	LR	0.93	0.39	0.83	0.87	0.56	0.82(0.69–0.95)
	RF	0.97	0.08	0.80	0.82	0.33	0.80.(0.66–0.94)
	SVM	1.00	0.00	0.81	0.81	1.00	0.72 (0.53–0.92)

Note.—Data in parentheses are 95% CIs. PPV = positive predictive value, NPV = negative predictive value, AUC = area under the receiver operating characteristic curve, LR = logistic regression, RF = Random Forest, SVM = Support Vector Machine

### Nomogram

**Fig. 5:**
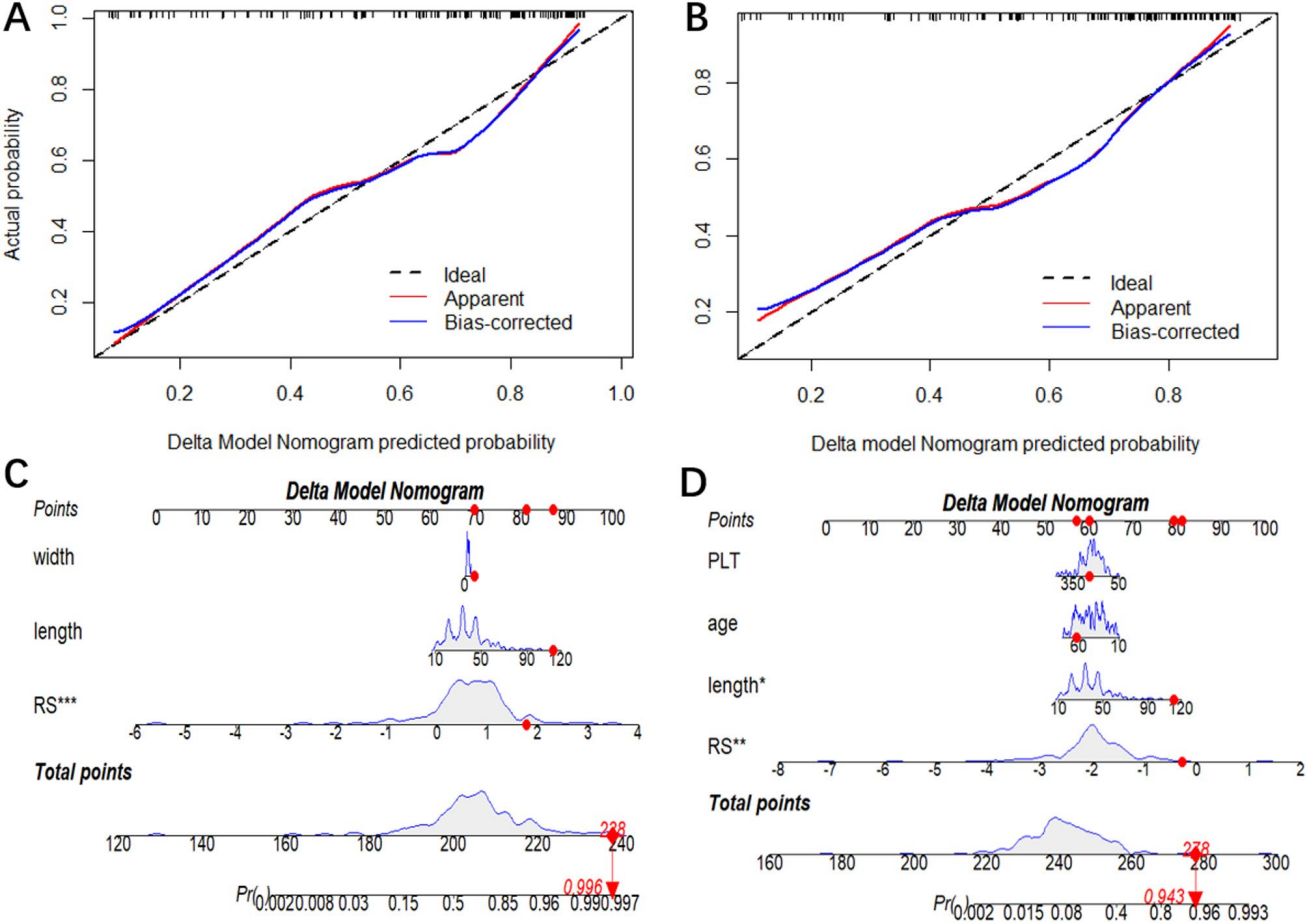
Calibration curves and Nomogram of the Delta model for predicting Intraoperative transfusion (**A**, **C**) and Intraoperative massive bleeding (**B**, **D**)

**Fig. 6:**
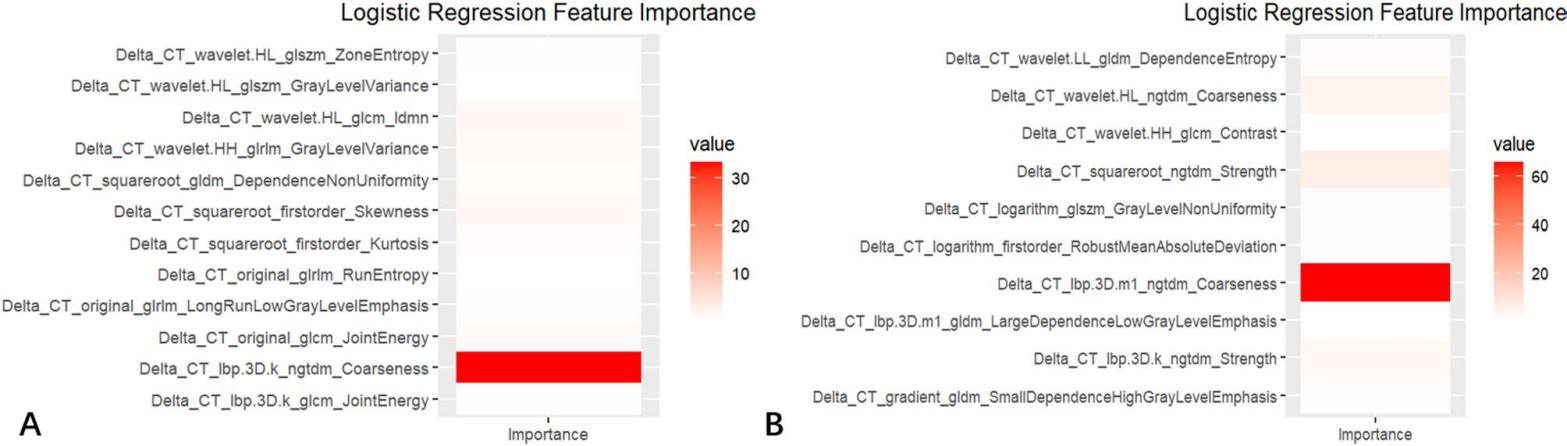
Importance of logistic regression features of the delta model for predicting intraoperative transfusion (**A**) and intraoperative massive bleeding (**B**)

Because of its advantages, such as ease of implementation and understanding, stable performance, and predictive ability superiority over that of other classifiers, LR was chosen to establish the nomogram to provide individualized probability estimates and weights of individual features [20, 22]. Nomograms created using the delta models to predict IBT and IMB are presented in Fig. 5. Calibration curves (all Brier scores < 0.25) also indicated that the predicted probabilities of IBT and IMB in the delta model did not differ significantly from the actual probabilities (*p* = 0.23, *p* = 0.35, *p*>0.05, respectively) (Fig. 5**)**. Additionally, the visualization of importance of the radiomics features in the LR-constructed delta model revealed that the radiomics feature Delta_CT_lbp.3D.k_ngtdm_Coarseness was significantly important (Fig. 6).

## Discussion

Preoperative identification of the risk of IBT and IMB is critical for patient prognosis, surgical planning and blood management. In our study, we developed two risk prediction models: the IBT model and the IMB model. Among these, the delta model performed best in predicting IBT and IMB risk, with better predictive ability than the clinical model (IDI = 0.11–0.13 for IBT, IDI = 0.02–0.08 for IMB, *p* < 0.05, respectively). It may be used as a valuable tool to improve risk stratification before spinal tumours surgery, thus contributing to preoperative planning and improving patient prognosis.

In the clinical variables, both IBT and IMB models identified tumor size (maximum axial length and vertical short diameter) as a risk factor. This suggests that tumor size not only directly reflects the tumor’s biological behavior and vascularity but also serves as a key determinant of surgical complexity. Larger tumors have a greater anatomical extent, increased blood supply, longer surgical duration, and higher surgical difficulty, all of which increase the risk of IBT and IMB [3, 23, 24]. This highlights the necessity for enhanced intraoperative monitoring in patients with larger spinal tumors and the consideration of advanced techniques, such as preoperative embolization or blood conservation strategies, to reduce the risk of excessive bleeding.

Furthermore, preoperative platelet count and patient age are significant predictors of IMB risk. On one hand, a low platelet count can indicate underlying issues that impair clot formation and increase bleeding risk. On the other hand, as patients age, the physiological reserve of organ systems declines, and comorbidities may exacerbate bleeding tendencies [5, 25, 26]. Therefore, nutritional support, age-appropriate management, and platelet optimization should be considered integral components of a comprehensive strategy to improve surgical outcomes and minimize IMB.

Contrast-enhanced CT was chosen as the imaging method for preoperative assessment of tumour blood supply and peritumour vascular distribution in this study for the following reasons: (1) CT volume imaging, which is more reflective of the real situation of tumour blood supply [27], (2) CT scanning has a consistent scanning range in all phases, which leads to better image registration, and (3) while DCE-MRI is usually expensive, the duration of the examination is long, and the patient is often results in poor image quality due to patient discomfort [28].

The delta model performed best in predicting IBT and IMB risk, with better predictive ability than the clinical model (IDI = 0.11–0.13 for IBT, IDI = 0.02–0.08 for IMB, *p* < 0.05, respectively), which demonstrates that the delta model better reflects the tumor vascularity, perfusion, and tissue heterogeneity of spinal tumors. Previous studies [5, 29] have proposed artificial intelligence-based prediction models for massive intraoperative blood loss in patients with metastatic spinal disease, but these models only included clinical variables, leading to somewhat inferior performance. Therefore, we believe that delta radiomics provides an added benefit in predicting IBT and IMB risk in patients with spinal tumors.

Additionally, we found that Delta_CT_lbp.3D.k_ngtdm_Coarseness plays an important role in predicting intraoperative transfusion and massive bleeding. This feature measures the texture of the tumor, specifically focusing on its “coarseness,” which reflects the heterogeneity of the tumor’s structure [30–32]. Tumors with coarser textures typically have irregular blood vessels, areas of necrosis, or fibrosis, all of which contribute to increased blood supply and fragile vasculature. These characteristics make the tumor more prone to bleeding during surgery. By capturing three-dimensional texture variations, this feature provides a more accurate representation of the tumor’s complexity, including its relationship with surrounding blood vessels [33, 34]. Therefore, Delta_CT_lbp.3D.k_ngtdm_Coarseness may be potential imaging biomarkers for identifying tumors with a higher risk of intraoperative bleeding and can help guide surgical planning and blood management strategies.

In addition, the development of delta models used three classifiers, including LR, RF and SVM, and the performance of each classifier has been relatively stable on the test set. In addition, to ensure the reliability of modeling, the 1:10 rule (i.e., approximately one feature can be studied per 10 events) was strictly followed when using logistic regression to analyze data in the training set using 10-fold cross-validation [35]. The use of 10-fold cross-validation helps reduce bias and variance, provides a comprehensive evaluation of the model, and enhances its generalizability to unseen data by ensuring that each data point is used for both training and test set. Finally, through nomogram visualizations, clinicians can easily perform preoperative assessment of the risks of IBT and IMB. Thus, the delta models we constructed can be valuable tools to preoperative identification of the risk of IBT and IMB.

Our study had some limitations. First, this study had inherent limitations because of its retrospective nature. For instance, the number of no-IMB patients was significantly higher than that of IMB patients, but we addressed this issue through data augmentation and balancing. Second, our study cohort were from a single center, and the predictive ability of our model requires further validation through external testing. Finally, factors such as surgical techniques and intraoperative practices significantly affect transfusion requirements and blood loss; however, we only considered preoperative factors.

## Conclusion

Intraoperative bleeding is a serious complication of spinal tumor surgery. Preoperative identification of the risk of intraoperative transfusion and massive bleeding is critical for patient prognosis, surgical planning and blood management. The CT delta model we constructed may be used as valuable tool to improve risk stratification before spinal tumour surgery, thus contributing to preoperative planning and improving patient prognosis.

## Electronic supplementary material

Below is the link to the electronic supplementary material.

Supplementary Material 1

## Data Availability

The datasets used and/or analysed during the current study are available from the corresponding author on reasonable request.

## Abbreviations

CT: Computed tomography
LR: Logistic regression
RF: Random forest
SVM: Support vector machine
AUC: Area under the receiver-operating characteristic curve
IDI: Integrated discrimination improvement
