# Supplementary material for: CT delta-radiomics predicts the risks of blood transfusion and massive bleeding during spinal tumor surgery
Source: Cancer Imaging. 2025 Jun 22;25:79. doi: 10.1186/s40644-025-00900-1 (PMC12183832; doi:10.1186/s40644-025-00900-1)
Supplement: Supplementary file 1 — Supplementary Material 1 [file 40644_2025_900_MOESM1_ESM.docx]

**Supplementary material 1. Imaging protocols**

All patients underwent unenhanced and contrast-enhanced multiphasic spine CT scans using one of the following systems: GE Revolution CT, SIEMENS SOMATOM Force, SIEMENS SOMATOM Definition Flash, UIH uCT 960+, UIH uCT 790, or UIH uCT 860. A 1.3–1.5 mL/kg body weight bolus of contrast material (iodixanol [Ultravist 370, Bayer, Germany] or ioversol [Optiray 320 or Optiray 350, Guerbet, France]) was injected intravenously at a flow rate of 2.0–3.5 mL/sec. Arterial phase scanning was initiated after a 25–35-second delay following enhancement of the descending aorta to 100 HU, as measured using a bolus-tracking technique. Imaging was conducted with consistent parameters for each patient.

| **Scanning parameters** | **Cervical** | **Thoracic** | | **Lumbar** |
| --- | --- | --- | --- | --- |
| Tube voltage (kVp) | 100 or 120 | 100 or 120 | | 100 or 120 |
| Tube current (mAs) | 60‐380 | 60‐380 | | 60‐380 |
| Field of view (mm) | 150 × 150–500 × 500 | 150 × 150–500 × 500 | 150 × 150–500 × 500 | |
| Matrix | 512 × 512 | 512 × 512 | | 512 × 512 |
| Slice thickness/pitch (mm) | 1 × 1 | 1 × 1 | | 1 × 1 |
| Delay time (s) | 25 | 30 | | 35 |

**Supplementary material 2: The packages of R software used for the statistical analysis**

The diagnostic performance of the three models was evaluated using receiver operating characteristic (ROC) curves, the area under the curve (AUC) was calculated, and the diagnostic performance, expressed by the AUC, was compared between models with the DeLong test. Calibration curves and the Hosmer-Lemeshow test were used to assess the agreement between the nomogram-predicted probability and the actual results of the risks of blood transfusion and massive bleeding during spinal tumor surgery.

Student's t-tests, Chi-Squared Tests, Mann–Whitney U-tests, and Kruskal-Wallis rank sum tests were done using the “stats”, “dplyr” and “broom” packages. Levene's test was done using the “car” package. The Hosmer-Lemeshow test was done using the “Resource Selection” package. Model building and feature selection using the “caret” package. Lasso binary logistic regression was done using the “glmnet” package. Support vector machine was done using the “e1071” package. Random forest was done using the “randomForest” package. Feature importance was done using the “Matrix”, and “reshape2” packages. Nomograms and calibration curves were done with the “rms” package. C-index calculation was performed using the “Hmisc” package. DeLong test and ROC curves plot was done using the “pROC” package. Integrated discrimination improvement was performed using the“PredictABE” package.

**Supplementary** **material 3**. Baseline Patient Characteristics

| **Characteristic** | **Training Set(n=161)** | **Test Set(n=70)** |
| --- | --- | --- |
| Patient demographics |  |  |
| Age (y) * | 46 (32-59) | 43 (28-56) |
| Sex(Male) | 89 (55) | 34 (49) |
| Clinical parameters |  |  |
| HB level (U/L) * | 140 (128-150) | 138 (130-150) |
| ALB level (U/L) * | 43.9 (42.0-46.2) | 44.0 (41.7-43.6) |
| PLT level (U/L) * | 233 (190-275) | 230 (192-251) |
| PE(Yes) | 37(23) | 11(16) |
| Tumor type(Primary) | 126 (78) | 58(83) |
| Pathological type(Benign) | 76 (47) | 41(59) |
| Radiologic features |  |  |
| Tumor location(Lumbosacral) | 30 (19) | 12 (17) |
| Tumor involved segment(Single) | 85 (53) | 37 (53) |
| Maximum long diameter (mm) * | 36 (26-46) | 38.5 (29-45) |
| Vertical short diameter (mm) * | 33 (23-44) | 33 (23-42) |
| Label |  |  |
| intraoperative transfusion(Yes) | 103 (64) | 43 (61) |
| Intraoperative massive bleeding(Yes) | 22 (14) | 13 (19) |

Note.—Unless otherwise indicated, data are numbers of patients, and data in parentheses are percentages.

HB=Hemoglobin, ALB=albumin, PLT=platelet, PE=Preoperative embolization.

* Data are medians, with IQRs in parentheses.

**Supplementary material 4** The radiomics selection process and correlation analysis between radiomics features and intraoperative blood transfusion.

**1. CT model:** Of the 1,688 radiomic features, 1,327 had good reproducibility (ICC >0.8) and were included in the feature selection step. A total of 884 features were retained after normalized and removed batch effects, 161 features were obtained after the Student’s t-tests, 13 were retained after redundancy analysis, and finally, 10 features were retained after LASSO regression and 10-fold cross-validation, comprising 10 texture features (1 from GLCM, 1 from GLRLM, 4 from GLSZM, and 4 from GLDM).

**2. CTE model:** Of the 1,688 radiomic features, 1,123 had good reproducibility (ICC >0.8) and were included in the feature selection step. A total of 563 features were retained after normalized and removed batch effects, 460 features were obtained after the Student’s t-tests, 13 were retained after redundancy analysis, and finally, 8 features were retained after LASSO regression and 10-fold cross-validation, comprising 10 texture features (2 from GLCM, 2 from GLSZM, 1 from GLDM), 2 first-order features, and 1 shape feature.

**3. Delta model:** Of the 1,688 radiomic features, 1,313 had good reproducibility (ICC >0.8) and were included in the feature selection step. A total of 674 features were retained after normalized and removed batch effects, 80 features were obtained after the Student’s t-tests, 27 were retained after redundancy analysis, and finally, 12 features were retained after LASSO regression and 10-fold cross-validation, comprising 10 texture features (3 from GLCM, 3 from GLRLM, 2 from GLSZM, 1 from GLDM, and 1 from NGTDM) and 2 first-order features.

**4. Results of correlation analysis between radiomics features and intraoperative blood transfusion of three models in the training set.**

| **Radiomics features** | correlation coefficient |
| --- | --- |
| CT_wavelet.HH_glszm_LowGrayLevelZoneEmphasis | 0.29 |
| CT_wavelet.HL_glszm_ZoneEntropy | 0.49 |
| CT_lbp.3D.m1_gldm_DependenceEntropy | 0.02 |
| CT_squareroot_glcm_MCC | 0.05 |
| CT_gradient_glszm_ZoneEntropy | 0.42 |
| CT_original_glrlm_LongRunEmphasis | 0.48 |
| CT_lbp.3D.k_gldm_GrayLevelVariance | 0.09 |
| CT_squareroot_gldm_LowGrayLevelEmphasis | -0.29 |
| CTE_original_shape_Elongation | 0.19 |
| CTE_lbp.3D.k_gldm_SmallDependenceLowGrayLevelEmphasis | -0.22 |
| CTE_lbp.3D.k_glcm_Imc1 | 0.61 |
| CTE_wavelet.HH_glszm_ZoneEntropy | -0.16 |
| CTE_squareroot_glcm_Imc2 | 0.44 |
| CTE_lbp.3D.k_firstorder_Mean | -0.05 |
| CTE_wavelet.LL_firstorder_Variance | 0.38 |
| CTE_squareroot_glszm_ZonePercentage | -0.4 |
| Delta_CT_lbp.3D.k_ngtdm_Coarseness | 0.07 |
| Delta_CT_wavelet.HL_glszm_ZoneEntropy | 0.01 |
| Delta_CT_wavelet.HL_glcm_Idm | -0.53 |
| Delta_CT_squareroot_firstorder_Skewness | 0.68 |
| Delta_CT_wavelet.HH_glrlm_GrayLevelVariance | 0.39 |
| Delta_CT_original_glcm_JointEnergy | 0.40 |
| Delta_CT_wavelet.HL_glszm_GrayLevelVariance | 0.08 |
| Delta_CT_squareroot_gldm_DependenceNonUniformity | -0.16 |
| Delta_CT_original_glrlm_LongRunLowGrayLevelEmphasis | 0.17 |
| Delta_CT_squareroot_firstorder_Kurtosis | -0.19 |
| Delta_CT_lbp.3D.k_glcm_JointEnergy | 0.02 |

**Supplementary material 5** The radiomics selection process and correlation analysis between radiomics features and intraoperative massive bleeding.

**1. CT model:** Of the 1,688 radiomic features, 1,234 had good reproducibility (ICC >0.8) and were included in the feature selection step. A total of 872 features were retained after normalized and removed batch effects, 516 features were obtained after the Student’s t-tests, 19 were retained after redundancy analysis, and finally, 4 features were retained after LASSO regression and 10-fold cross-validation, comprising 1 texture feature (1 from GLSZM), 2 first-order features, and 1 shape feature.

**2. CTE model:** Of the 1,688 radiomic features, 1,045 had good reproducibility (ICC >0.8) and were included in the feature selection step. A total of 952 features were retained after normalized and removed batch effects, 606 features were obtained after the Student’s t-tests, 32 were retained after redundancy analysis, and finally, 6 features were retained after LASSO regression and 10-fold cross-validation, comprising 3 texture features (3 from GLSZM) and 3 first-order features.

**3. Delta model:** Of the 1,688 radiomic features, 956 had good reproducibility (ICC >0.8) and were included in the feature selection step. A total of 342 features were retained after normalized and removed batch effects, 39 features were obtained after the Student’s t-tests, 11 were retained after redundancy analysis, and finally, 10 features were retained after LASSO regression and 10-fold cross-validation, comprising 9 texture features (1 from GLCM, 1 from GLSZM, 3 from GLDM, and 4 from NGTDM) and 1 first-order features.

**4. Results of correlation analysis between radiomics features and intraoperative blood transfusion of three models in the training set.**

| **Radiomics features** | **correlation coefficient** |
| --- | --- |
| CT_original_shape_Flatness | 0.23 |
| CT_logarithm_glszm_ZoneEntropy | 0.29 |
| CT_squareroot_firstorder_Range | 0.16 |
| CT_lbp.3D.m1_firstorder_Energy | 0.37 |
| CTE_wavelet.LL_firstorder_Range | 0.14 |
| CTE_exponential_glszm_SmallAreaEmphasis | -0.09 |
| CTE_wavelet.HL_firstorder_Maximum | 0.04 |
| CTE_wavelet.HH_glszm_GrayLevelVariance | -0.04 |
| CTE_gradient_glszm_ZoneEntropy | 0.03 |
| CTE_lbp.3D.m2_firstorder_TotalEnergy | 0.33 |
| Delta_CT_gradient_gldm_SmallDependenceHighGrayLevelEmphasis | 0.48 |
| Delta_CT_logarithm_glszm_GrayLevelNonUniformity | -0.96 |
| Delta_CT_logarithm_firstorder_RobustMeanAbsoluteDeviation | -0.90 |
| Delta_CT_wavelet.HL_ngtdm_Coarseness | -1.68 |
| Delta_CT_lbp.3D.m1_gldm_LargeDependenceLowGrayLevelEmphasis | 0.37 |
| Delta_CT_wavelet.LL_gldm_DependenceEntropy | -0.93 |
| Delta_CT_squareroot_ngtdm_Strength | 3.76 |
| Delta_CT_lbp.3D.k_ngtdm_Strength | -1.25 |
| Delta_CT_lbp.3D.m1_ngtdm_Coarseness | -48.63 |
| Delta_CT_wavelet.HH_glcm_Contrast | 0.47 |
